# Distribution of glucagon-like peptide 1 receptor and insulin in phaeochromocytomas

**DOI:** 10.1530/EC-25-0338

**Published:** 2026-01-13

**Authors:** Ivar Følling, Maria Lie Selle, Hogne Røed Nilsen, Anna Berit Wennerström, Hilde Loge Nilsen, Tor Jacob Eide

**Affiliations:** ^1^ Department of Endocrinology, Akershus University Hospital, Lørenskog, Norway; ^2^ Insitute of Clinical Medicine, University of Oslo, Oslo, Norway; ^3^ Health Services Research Unit, Akershus University Hospital, Lørenskog, Norway; ^4^ Division of Laboratory Medicine, Department of Pathology, Oslo University Hospital, Oslo, Norway; ^5^ Department of Clinical Molecular Biology, University of Oslo and Akershus University Hospital, Lørenskog, Norway; ^6^ Department of Microbiology, Oslo University Hospital, Oslo, Norway; ^7^ CRESCO – Centre for Embryology and Healthy Development, University of Oslo, Oslo, Norway

**Keywords:** glucagon-like peptide 1 receptor, insulin, tumour-induced hypoglycaemia, phaeochromocytoma

## Abstract

**Introduction:**

Most phaeochromocytomas produce insulin, and some produce glucagon-like peptide 1 receptor (GLP-1R). In pancreatic β-cells, stimulation of GLP-1R causes insulin release. A few phaeochromocytoma patients experience hypoglycaemic attacks. Therefore, we studied the distribution of GLP-1R-containing and insulin-containing phaeochromocytoma cells and their relation.

**Methods:**

In 20 phaeochromocytomas, we performed sequential double staining with anti-insulin and anti-GLP-1R antibodies and, in selected cases, staining with anti-insulin alone. We quantified tumour cells with positive staining and compared their distribution to that of randomly distributed cells using simulations. We obtained GLP-1R transcript data from 182 such tumours from The Cancer Genome Atlas (TCGA) Research Network.

**Results:**

GLP-1R-containing cells were found in six of the 20 tumours, and insulin-containing cells were found in fifteen. Moreover, in the TCGA cohort, almost half of the tumours produce GLP-1R transcripts, and patients with the highest number of transcripts show longer disease-free survival. In the tumours, we found that cells expressing insulin were present in the cytoplasm and GLP-1R in the membrane, with a frequency of 2.59 and 1.34%, respectively. These cells showed clustering, and one tumour showed a large clonal expansion. Interestingly, we found deposits of insulin, which we suggest naming insulin bodies in two tumours. Very few cells contained both proteins.

**Conclusion:**

Most phaeochromocytomas contain tumour cells producing insulin. About half produce GLP-1R. The producing cells show clustering, and clonal expansion occurs. Insulin release might cause hypoglycaemia. Increased GLP-1R levels might induce less aggressive tumours.

## Introduction

Phaeochromocytomas produce epinephrine and norepinephrine, and some, in addition, produce ectopic hormones. Most phaeochromocytomas show ectopic production of insulin (1, 2), insulin transcript and a related hybrid transcript (2). In most of these tumours, only few cells produce insulin, but in some of the tumours, insulin-containing cells form clusters, indicating clonal expansion (2).

If such tumours release insulin to the blood, it could explain the rare and unexplained occurrence of hypoglycaemic attacks described in a few patients with phaeochromocytoma (3, 4, 5, 6); in at least two of them, the hypoglycaemic attacks were hyperinsulinaemic (4, 5) and one was cured from such attacks by removal of the tumour (5).

Stimulation of the glucagon-like peptide 1 receptor (GLP-1R) in the membrane of pancreatic β-cells causes insulin release from these cells. We hypothesized that a similar release mechanism occurs in phaeochromocytomas.

It is known that some phaeochromocytomas contain GLP-1R (7, 8, 9, 10). However, no report has described the distribution of this GLP-1R or if it co-localizes with insulin-containing cells, necessary for this type of insulin release. Therefore, in this study, we describe in detail the distribution of GLP-1R-containing cells and insulin-containing cells and their relation.

## Methods

### Patients and tumours

Informed consent was obtained from 20 patients with phaeochromocytomas operated consecutively, to retrospectively investigate their tumours (2). In brief, they constitute a typical cohort of phaeochromocytoma patients. Clinical and laboratory data are presented in Supplementary Table 1 (see section on Supplementary materials given at the end of the article). There are 10 of each sex (age span 30–70 years), of which two had germline mutations (one heterozygous FN1 mutation: c.1527+4_1527+7 del; one MAX mutation: exon 3 c.149>A (Val 50 Asp)) predisposing to phaeochromocytoma and seven had hyperglycaemia. Data of PASS (11), plasma metanephrines, and findings of GLP-1R and insulin in the tumours are given. No patient showed signs of metastases, and all PASS values were below 5, indicating benign tumours. Tumour diameters were 1.9–9.5 cm (median 5 cm).

### Ethical approval

Consent was given by the Regional Ethics committee (REC South-East Norway) (approval no. 2018/196) and the Akershus University Hospital Data Protection Office (approval no. 7-2018).

### Immunohistochemistry

Detection of GLP-1R and insulin was performed by doing sequential double staining on 4 μm formalin-fixed paraffin-embedded sections using the automated Ventana Discovery Ultra system (Roche, Norway, Cat No. 05987750001).

To prepare the tissue section prior to staining, we used Discovery CC1 (Roche, Cat No. 06414575001) for antigen retrieval for 48 min, followed by inhibition of endogenous peroxidase using Discovery Inhibitor (Roche, Cat No. 07017944001) for 4 min.

Staining: rabbit monoclonal anti-GLP-1R (EPR23507-57) (Abcam, UK, Cat No. ab254352) at dilution 1:25 was incubated for 48 min at 37°C followed by Discovery OmniMap anti-Rabbit HRP (Roche, Cat No. 05269679001) and Discovery Purple Kit (Roche, Cat No. 07053983001). Mouse monoclonal anti-insulin (2D11-H5) (Invitrogen, USA, Cat No. MA1-35525) at dilution 1:400 was incubated for 32 min at 37°C, followed by Discovery OmniMap anti-Mouse HRP (Roche, Cat No. 05269652001) and Discovery Teal HRP Kit (Roche, Cat No. 08254338001). For denaturation and neutralizing between the anti-GLP-1R and the anti-insulin sequences, Ultra CC2 (Roche, Cat No. 05424542001) and Discovery Inhibitor (Roche, Cat No. 07017944001) were used. Sections were counterstained in Shandon Instant Hematoxylin (Fisher Scientific, Norway, Cat. No. 6765015) for 1 min and mounted with Eukitt (Sigma-Aldrich, Norway, Cat. No. 03989). In addition, chromogen insulin staining (2) was included for selected images because of improved contrast between insulin-containing cells (brown) and the other tumour cells.

### Distribution of cells containing GLP-1R and cells containing insulin

Of the 20 tumours, fifteen showed cells containing insulin, and in six, we found cells containing GLP-1R. We studied the distribution of these cells in detail in the following.

Evaluation of the number of tumour cells displaying positivity for GLP-1R relative to the total number of tumour cells was performed by randomly selecting 20 areas with photo documentation from each case by using a Nikon C-TEP3 mounted camera on a Nikon eCLIPSE C*i* microscope with the use of a Nikon Plan-Apo 20×/0.75 λ objective. The cross-board was moved within the border lines of the tumour-tissue marked with a coloured pen on the slides. Each printed photo was evaluated both for the total number of tumour cells and for tumour cells positive for GLP-1R. Positivity for GLP-1R was defined when at least one membranous line along one side of a tumour cell was observed with purple colour. The number of tumour cells on all 20 photos and the corresponding number of tumour cells positive for GLP-1R cells were used for calculating the percentage of GLP-1R-positive cells in each of the six tumours. We used the same procedure for cells containing insulin, with the exception that we selected 10 areas per tumour instead of 20, due to the increased quantity of insulin-containing cells. Positivity for insulin was defined when a substantial part of the cytoplasm of the tumour cells was marked with turquoise colour, and similar calculations were made.

### Bioinformatics

Gene expression data from phaeochromocytoma and paraganglioma (PCPG) are available through The Cancer Genome Atlas (TCGA) Research Network (https://www.cancer.gov/ccg/research/genome-sequencing/tcga). The PCPG cohort consists of data from 182 tumours that were molecularly and clinically described previously (12). The differential gene expression analysis comparing expression (cut-off value of 1.5) in the PCPG tumours with the normal controls was performed by ANOVA in the online analysis tool Gene Expression Profiling Interactive Analysis (GEPIA) (13).

### Statistics

To confirm whether GLP-1R-containing cells or insulin cells were clustered, a nearest-neighbour analysis was performed using the Clark–Evans R index (14). This index compares the observed average distance between each GLP-1R-positive cell or insulin cell and its nearest neighbour to the expected distance under complete spatial randomness, modelled as a homogeneous Poisson point process. A value of *R* below 1 indicates clustering, meaning that cells are located closer together than expected by chance. A total of 500 homogeneous Poisson point process fields were simulated, using the dimensions of the visual fields from the tumour slices and the average number of GLP-1R-containing cells or insulin cells as cell intensity. Statistical significance of clustering was then determined by comparing the observed Clark–Evans R index to the distribution of *R* values obtained from 500 simulated homogeneous Poisson point patterns. The two-sample Kolmogorov–Smirnov test was used to compare the empirical nearest neighbour distance distribution of GLP-1R-positive cells to that of insulin cells, to test whether there was significantly more clustering for GLP-1R-containing cells than for insulin cells.

## Results

Double immunohistochemical staining was performed on each of the 20 phaeochromocytomas. Tumours were co-stained with anti-GLP-1R and anti-insulin antibodies, thus enabling us to observe an eventually mutual relationship between the two proteins in the tumour tissue.

First, we confirmed previous findings (2). Of the 20 phaeochromocytomas, 15 show tumour cells producing insulin. In most tumours, there are few such cells. In some tumours, these cells show clustering, suggesting clonal expansion. Six tumours show cells containing GLP-1R, also with clustering.

**Figure 1:**
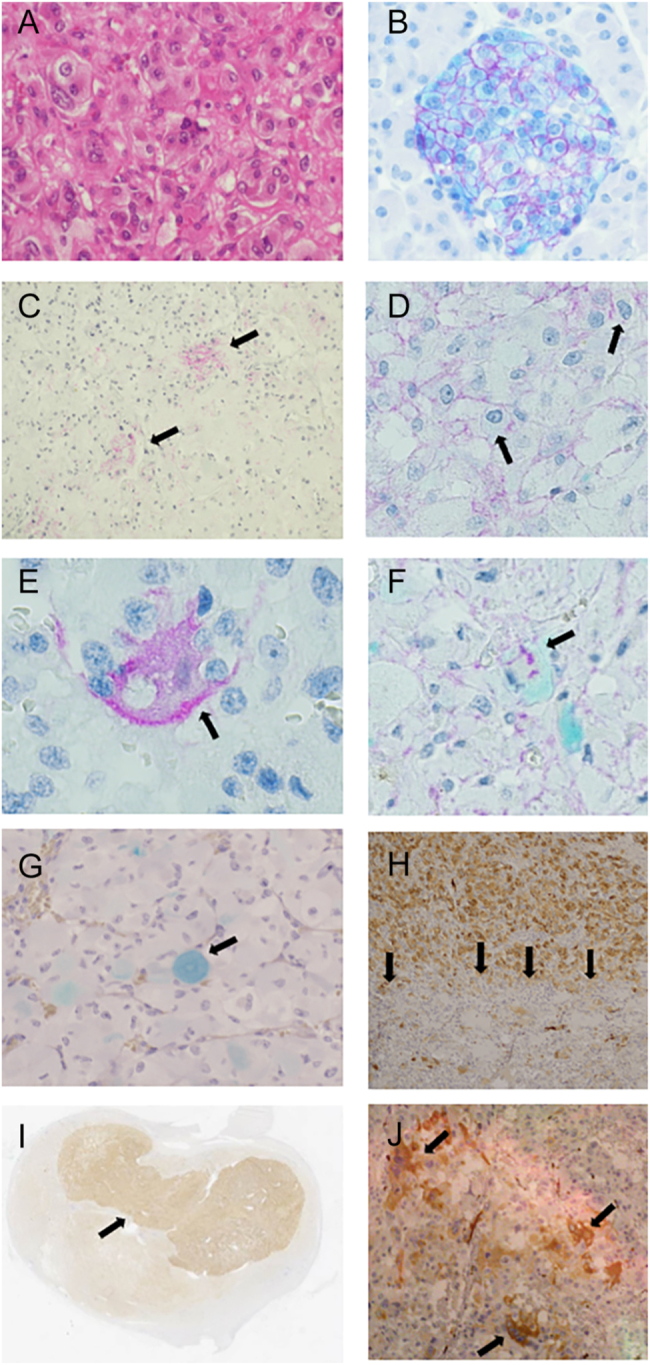
Phaeochromocytomas stained with monoclonal anti-GLP-1R and monoclonal anti-insulin antibodies. (A, B, C, D, E, F, G) GLP-1R appears purple, and insulin appears turquoise. (A) Standard haematoxylin–eosin staining showing a typical phaeochromocytoma. (B) Normal pancreas with islet. GLP-1R is located in the whole membrane, and insulin is located in the cytoplasm of the islet β-cells. The exocrine tissue serves as a negative control. (C) Groups of GLP-1R-positive phaeochromocytoma cells (arrows) show that these cells do not spread randomly. They tend to appear in clusters. (D) GLP-1R often localizes in patches in the membrane (arrows), different from distribution in the whole membrane of the β-cells. (E) In a few cells, GLP-1R is also found in the cytoplasm (arrow). (F) A phaeochromocytoma cell (arrow) containing both GLP-1R and insulin. This occurs rarely as expected from the number of positive cells if the two proteins occur independently. (G) Insulin body (arrow). (H, I, J) Insulin appears brown. (H) A large clonal expansion (upper half) packed with insulin-containing cells, with only few insulin-containing cells in the lower half. Border indicated with arrows. (I) Macroscopic view of (H) showing that the darker upper half (arrow), the clonal expansion, constitutes around half of the tumour. (J) Small clusters of insulin-containing cells (arrows).

Conventional haematoxylin–eosin staining of the tumours revealed typical phaeochromocytoma histology (Fig. 1A). A normal pancreatic endocrine island with an abundance of β-cells surrounded by the exocrine tissue (Fig. 1B). These cells contain insulin (turquoise) in the cytoplasm and GLP-1R (purple) in the membrane (Fig. 1B). GLP-1R constitutes a continuous band in the membrane around the whole β-cell. The exocrine tissue serves as a negative control, with no staining for insulin or GLP-1R. Normal adrenal medulla, where phaeochromocytomas originate, also does not stain for GLP-1R or insulin.

In the tumours, we found that GLP-1R was localized in the cell membrane and insulin in the cytoplasm, as in the pancreatic β-cells, with clusters of GLP-1R-containing cells (Fig. 1C). We found this tendency to cluster formation of GLP-1R-containing cells in all six tumours.

**Figure 2:**
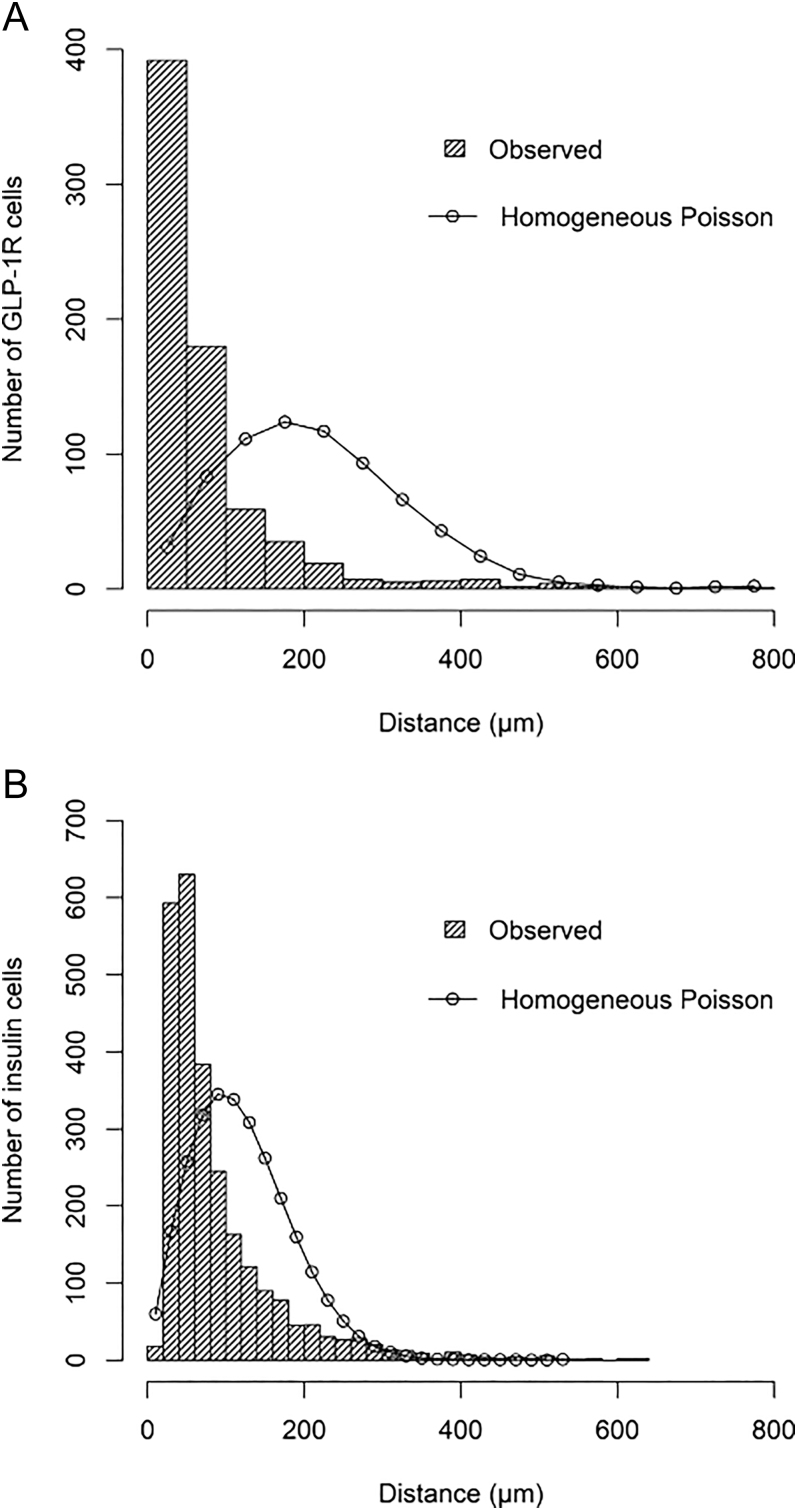
Spatial distribution of GLP-1R-containing cells and insulin-containing cells. Observed distances to the nearest GLP-1R-containing cell (A) and to the nearest insulin cell (B) for all tumours together. Both are significantly less than the simulated distances assuming a homogenous random Poisson distribution, indicating clustering (*P* < 0.001).

The clustering of insulin-containing cells is similarly shown visually in Fig. 1J and statistically in Fig. 2B and in Table 3.

Many of the GLP-1R-containing cells showed GLP-1R staining, which appeared patchier than the continuous pattern in the normal β-cells (Fig. 1D). This holds for all six tumours.

Most of the tumours contain a few cells with GLP-1R also in the cytoplasm, indicating intracellular synthesis or receptor internalization (Fig. 1E). We observed only few cells showing both insulin in the cytoplasm and GLP-1R in the membrane (Fig. 1F), suggesting that the two proteins occur independently. In two tumours, we found deposits of insulin, 60–70 μm in diameter, which we suggest naming insulin bodies (Fig. 1G). One tumour contained a large clonal expansion of insulin-containing cells (Fig. 1H and I), as a second and different neoplastic expansion inside the main tumour. The border between them is sharp (Fig. 1H) and lined with lymphocytes not seen in the rest of the tumour, a lining typical for many benign and malignant tumours. This phaeochromocytoma was an extreme outlier in terms of the number of insulin-containing cells and was therefore not included in the tables or in the statistical calculations. The expansion occupied about half the size of the whole tumour (Fig. 1I), which had a diameter of 23 mm.

Interestingly, the GLP-1R-containing cells appeared more closely spaced than expected under a homogeneous Poisson distribution, indicating clustering (Fig. 2A). A similar pattern was found for insulin-containing cells, although with less pronounced clustering compared with the GLP-1R-containing cells (Fig. 2B). Both GLP-1R cells and insulin cells showed significant clustering as evaluated by Clark–Evans R indices; GLP-1R had a value of 0.37 (*P* < 0.001), while insulin had a value of 0.75 (*P* < 0.001). More pronounced clustering of GLP-1R-containing cells than insulin-containing cells (Fig. 2) was evident from the R indices (Table 1), and further confirmed with the Kolmogorov–Smirnov test (*P* < 0.001). Thus, the visual observations (Fig. 1C and J) were confirmed statistically.

**Table 1:** Clark–Evans R indices and associated *P*-values for GLP-1R-positive cells.

Tumour*	*R* ^†^	*P*-value
Global	0.37	<0.001
Tumour 3	0.11	<0.001
Tumour 5	0.22	<0.001
Tumour 8	0.68	<0.001
Tumour 10	0.31	<0.001
Tumour 12	0.34	<0.001
Tumour 18	0.40	<0.001

* Tumour numbers refer to patient numbers as given in Supplementary Table 1.

^†^ *R* value <1 indicates clustering.

When analysing the tumour slices separately for clustering, GLP-1R-positive cells showed significant clustering in all six tumours, with R indices consistently below 1 (*P* < 0.001) (Table 1). The insulin-positive cells showed significant clustering in 7 out of 13 tumours (*R* < 1 and *P* < 0.05) (Table 2). The statistical tests applied to the separate tumour slices were not adjusted for multiple testing. Applying Bonferroni correction for multiple testing did not change the significance of the R indices for GLP-1R cells, while the number of tumours showing significant clustering for insulin cells would be reduced from 7 out of 13 to 6 out of 13.

**Table 2:** Clark–Evans R indices and associated *P*-values for insulin-positive cells.

Tumour*	*R* ^†^	*P*-value
Global	0.75	**<0.001**
Tumour 2	0.79	**<0.001**
Tumour 3	Insufficient data	
Tumour 4	0.85	**0.026**
Tumour 5	0.96	0.195
Tumour 6	0.98	0.377
Tumour 8	0.91	0.243
Tumour 9	1.00	0.558
Tumour 10	0.53	**<0.001**
Tumour 12	0.84	0.051
Tumour 13	0.77	**<0.001**
Tumour 14	0.91	0.056
Tumour 15	0.81	**<0.001**
Tumour 17	0.85	**<0.001**
Tumour 20	0.64	**<0.001**

Bold indicates statistical significance, *P* < 0.05.

* Tumour numbers refer to patient numbers as given in Supplementary Table 1.

^†^ *R* value <1 indicates clustering.

The number and proportion of phaeochromocytoma cells that contain insulin or GLP-1R were scored in all six tumours together and for each tumour separately (Table 3). The average frequency of GLP-1R-containing cells was 1.34%, range 0.08–3.02%. The average frequency of insulin-containing cells was 2.59%, range 0–5.55%, slightly larger than that of GLP-1R cells, *P* < 0.001 according to a two-sided *z*-test on the proportions. Thus, in most of the tumours, relatively few tumour cells contain insulin or GLP-1R. There was no correlation between the clinical or laboratory data given in Supplementary Table 1 and the presence of insulin or GLP-1R.

**Table 3:** Tumour cells expressing insulin and GLP-1R*.

	Tumour cells		Insulin-positive cells			GLP-1R-positive cells		
	*n*	Range	*n*	Range	Proportion (%)	*n*	Range	Proportion (%)
All tumours	54,361	131–1,266	1,406	0–100	2.59	730	0–60	1.34
Tumour 3	11,794	297–1,266	70	0–35	0.59	9	0–3	0.08
Tumour 5	8,346	148–716	463	0–87	5.55	21	0–14	0.25
Tumour 8	10,150	340–690	380	0–87	3.74	263	0–48	2.59
Tumour 10	10,074	244–777	113	0–26	1.12	177	0–60	1.76
Tumour 12	7,973	208–571	381	0–100	4.78	78	0–9	0.98
Tumour 18	6,024	131–533	0	0–0	0.00	182	0–51	3.02

* Twenty visual fields were counted for each tumour. Tumour numbers refer to patient numbers as given in Supplementary Table 1.

Range shows the minimum and maximum values over all visual fields. Proportion shows how many of the tumour cells were stained with anti-insulin or anti-GLP-1R antibodies. GLP-1R = glucagon-like peptide 1 receptor.

To test whether GLP-1R and insulin cells happen to appear in the same regions of the tumour, the Spearman correlation between the number of insulin cells and GLP-1R cells in visual fields was computed. The correlation *R* = 0.14 (*P* = 0.13) indicates low correlation between the GLP-1R-containing and insulin-containing cells. If the two proteins, GLP-1R and insulin, occur independently in the tumour cells, one would expect the frequency of cells presenting both proteins to be very rare with frequency 1.34 × 2.59% = 0.035%. This is in line with the visual observation that this is a very rare event (Fig. 1F), which suggests independency.

### Expression of GLP-1R transcripts in phaeochromocytoma tumours validated in TCGA

**Figure 3:**
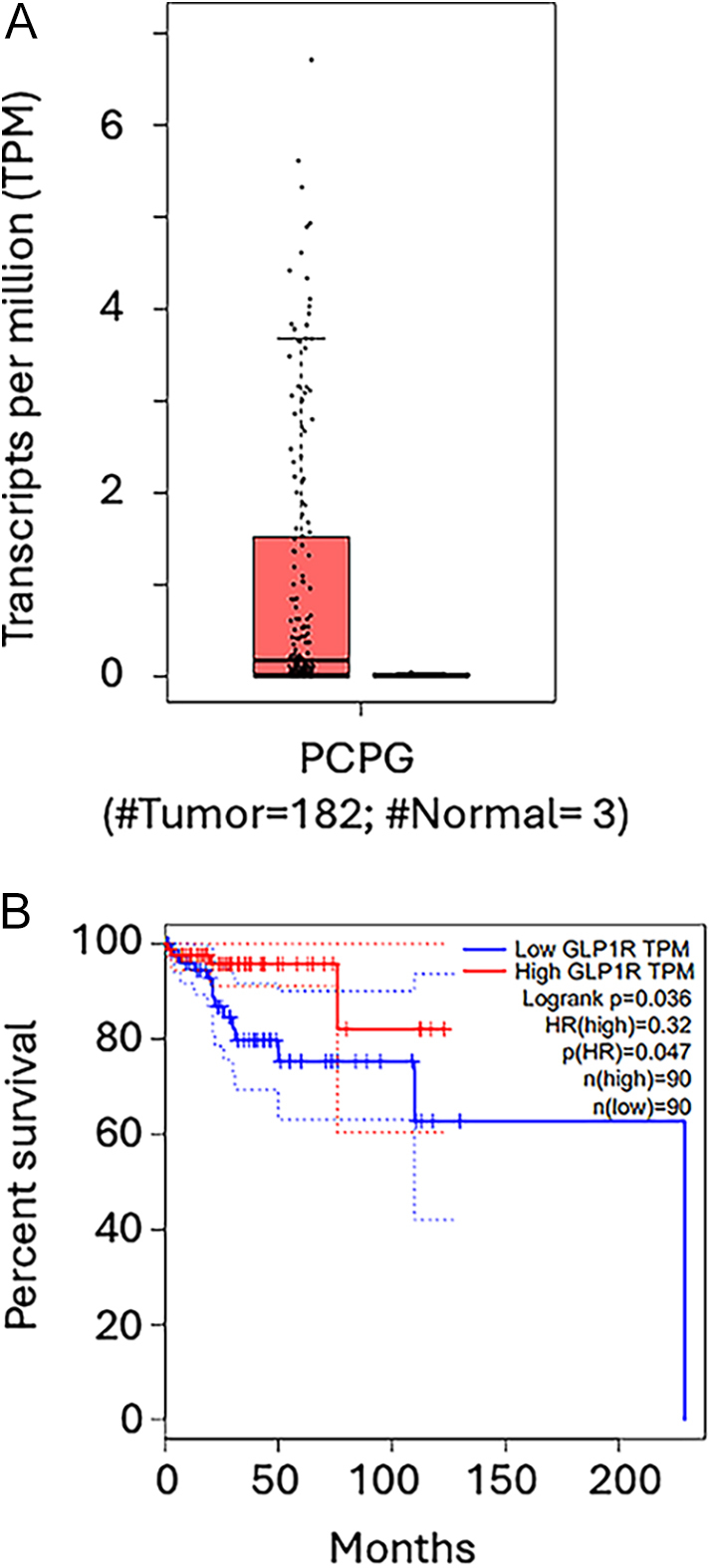
Expression of GLP-1R transcripts in phaeochromocytoma tumours. A TCGA cohort of 182 phaeochromocytomas and paragangliomas shows that (A) almost half of the tumours produce GLP-1R transcripts, whereas normal adrenal tissue does not. Cells synthesizing GLP-1R occur in many phaeochromocytomas. (B) Patients with tumours with a high number of GLP-1R transcripts show a significantly higher disease-free survival than those with a low number of transcripts, suggesting that the number of transcripts may have clinical implications.

To validate the transcription of GLP-1R in a larger cohort, we analysed a TCGA cohort of 182 phaeochromocytomas and paragangliomas. Figure 3A shows that almost half of the tumours produce such transcripts, whereas normal adrenal tissue does not. This corroborates our findings in that not only the protein but also the corresponding transcripts are present. Thus, cells synthesizing GLP-1R occur in many phaeochromocytomas.

Figure 3B suggests that this may have clinical implications in that patients with tumours with a high number of transcripts showed a significantly higher disease-free survival than those with a low number of transcripts.

## Discussion

Phaeochromocytomas produce epinephrine and norepinephrine. In addition, some of the tumours show ectopic production of hormones and proteins. Among those are insulin (1, 2) produced in some tumour cells in most phaeochromocytomas (2) and GLP-1R found in some phaeochromocytomas (7, 8, 9, 10). Here, we show the distribution and relation of these two proteins in phaeochromocytomas. Our main interest was to see if the two co-localize in the same cells so that stimulation of GLP-1R could cause insulin release, similar to the release from normal endocrine pancreatic β-cells. If so, it might explain the hypoglycaemic attacks seen in a few phaeochromocytoma patients (3, 4, 5, 6).

The reported frequencies of GLP-1R-producing phaeochromocytomas vary: five out of six (9) and 12 out of 20 (8), both with ligand receptor autoradiography; we found GLP-1R in six of the 20 tumours with immunohistochemistry and the corresponding transcripts in almost half of 182 tumours with RNA sequencing. The variation in frequencies can be due to differences between cohorts and between techniques used for detection. A reasonable estimate based on the collective findings is that about half of phaeochromocytomas produce GLP-1R.

GLP-1R sits mainly in the cell membrane, somewhat patchier than in β-cells. The GLP-1R-containing cells show clustering, documented by two independent methods: first, the visual observation and, second, the statistical finding of clustering.

The clustering can be due to clonal expansion, which may lead to areas where these cells dominate in larger parts of the tumour. Another explanation of clustering may be that in some parts of the tumour, the local structure could facilitate expression of GLP-1R. This seems less likely than clonal expansion, because we found no signs of altered tumour histology in areas where GLP-1R-containing cells are abundant.

The clustering of insulin-containing cells is similarly shown visually and statistically. The large clonal expansion strongly supports that clustering may progress to a large second and different neoplastic expansion inside the primary tumour. A rough calculation shows that a normal pancreas weighs around 80 g and the β-cells comprise 2% (15, 16), which gives 1.6 g of β-cells. The tumour diameter is 23 mm, which gives a weight of around 5 g, half of which is then packed with around 2.5 g of insulin-containing cells, more than in the whole of a normal pancreas. Therefore, the clonal expansion may contain more than enough insulin to cause hypoglycaemia, if released to the blood. Generally, the release from tumours with ectopic hormone production often lacks normal release control (17), also shown in insulin-producing tumours (18, 19). Thus, phaeochromocytomas may cause hypoglycaemia from insulin release also without stimulation of the GLP-1R.

The average number of GLP-1R-containing cells is low, 1.34% in the six tumours studied in detail. Insulin-containing cells are more frequent. They are present in most of the 20 tumours and in 2.59% of the cells in the tumours where we also find GLP-1R-containing cells. Thus, GLP-1R expression is a rarer event than the expression of insulin. Both the number of cells containing insulin or GLP-1R, as well as their appearance in clusters, may occur more frequently than our data describe, because there may be clonal expansions in other parts of the tumours than those we have investigated. The production of both proteins takes place inside the tumour cells, as insulin transcripts (2) and GLP-1R transcripts are found in the TCGA cohort.

Phaeochromocytomas show considerable heterogeneity, probably because different driver germline and somatic mutations, differences in gene expression, metabolic rewiring and epigenetic changes act together to shape the phenotype (20).

The clinical implications of our findings are uncertain. Few cells contained both GLP-1R and insulin, which is to be expected if the two proteins occur independently. This makes it unlikely that GLP-1R stimulation could cause the release of clinically significant amounts of insulin from a phaeochromocytoma. Our initial hypothesis of such a mechanism is therefore not supported by our findings. However, one cannot exclude that clonal expansion of cells containing both proteins could occur very rarely, greatly increasing the number of such cells, and cause GLP-1R-stimulated insulin release and hypoglycaemic episodes in phaeochromocytoma patients. Because of the rare occurrence of patients with hypoglycaemic fits, the mechanism could be a similarly rare occurrence of clonal expansion mentioned above.

The finding of a significantly higher disease-free survival in patients with high levels of GLP-1R transcripts than in those with low levels is interesting. However, this needs further verification. The mechanism could be that GLP-1R stimulation induces differentiation of phaeochromocytoma cells (10), which may mean less aggressive tumours.

To our knowledge, insulin bodies have not been described earlier. They should be considered as a new example of protein bodies seen in several other types of proliferative diseases. A typical example is Russell bodies containing immunoglobulins in plasma cell proliferations.

The strengths of our study are as follows: our observations of the occurrence and distribution of cells containing GLP-1R and insulin, their clustering and clonal expansion, and the insulin bodies expand the knowledge of the biology of phaeochromocytomas concerning ectopic production of proteins that are not present in the normal adrenal medulla. We discuss possible clinical consequences.

The limitations of our study are as follows: the main limitation is the number of cases studied. In the future, for phaeochromocytoma patients with hypoglycaemic attacks, it would be important to study insulin release before and after the removal of the tumour and perform detailed studies of the tumour. Future prospective studies of the ectopic production of insulin and GLP-1R in phaeochromocytomas should preferentially include a larger number of patients and perform detailed observations of clinical and laboratory data relating to GLP-1, GLP-1R, glucose metabolism, catecholamines and genetics, which we lack because our study is retrospective. *In vivo* experiments to test the production of insulin and GLP-1R would be of value. Our contribution is also restricted to benign phaeochromocytomas and does not include paragangliomas.

## Conclusion

Most phaeochromocytomas contain tumour cells producing insulin, and about half of phaeochromocytomas contain tumour cells producing GLP-1R. Insulin appears in the cytoplasm and GLP-1R appears in the membrane, as in pancreatic β-cells. In most tumours, both proteins appear in few of the tumour cells, and only very few cells contain both proteins. Both insulin-producing and GLP-1R-producing cells show clustering, and we find clonal expansion, which may give a greatly increased number of such cells in parts of the tumours.

The discovery of insulin bodies is a new observation. Clinical implications remain uncertain. Insulin release from a clonal expansion, with or without GLP-1R stimulation, might cause hypoglycaemia, and increased levels of GLP-1R might induce less aggressive tumours.

## Supplementary materials



## Declaration of interest

The authors declare that there is no conflict of interest that could be perceived as prejudicing the impartiality of this work.

## Funding

This work was partially supported by the Research Council of Norway through its Centres of Excellence scheme, project number 332713.

## Ethical approval

Consent was given by the Regional Ethics committee (REC South-East Norway) (approval no. 2018/196) and the Akershus University Hospital Data Protection Office (approval no. 7-2018).

## References

[1] Kamio T, Shigematsu K, Kawai K, et al. Immunoreactivity and receptor expression of insulinlike growth factor I and insulin in human adrenal tumors. An immunohistochemical study of 94 cases. Am J Pathol 1991 138 83–91. 1702931 PMC1886050

[2] Følling I, Wennerström AB, Eide TJ, et al. Phaeochromocytomas overexpress insulin transcript and produce insulin. Endocr Connect 2021 10 815–824. (10.1530/ec-21-0269) 34170845 PMC8346199

[3] Abdulhadi B, Anastasopoulou C & Lekprasert P. Tumor-induced hypoglycemia: an unusual case report and review of literature. AACE Clin Case Rep 2021 7 80–83. (10.1016/j.aace.2020.11.002) 33851027 PMC7924146

[4] Frankton S, Baithun S, Husain E, et al. Phaeochromocytoma crisis presenting with profound hypoglycaemia and subsequent hypertension. Hormones 2009 8 65–70. (10.14310/horm.2002.1224) 19269923

[5] Følling I, Olsen A, Nermoen I, et al. Phaeochromocytoma and hypoglycaemic fits: a case report. Endocr Abstracts 2015 37 EP1156. (10.1530/endoabs.37.EP1156)

[6] Thonangi RP, Bhardwaj M & Kulshreshtha B. A case report of reactive hypoglycemia in a patient with pheochromocytoma and it’s review of literature. Indian J Endocrinol Metab 2014 18 234–237. (10.4103/2230-8210.129120) 24741525 PMC3987279

[7] Saber-Ayad M, Zaher D, Manzoor S, et al. Effect of proliferation and migration in pheochoromocytoma and colorectal cancer cells. ESMO Open 2018 3(Suppl2) A199. (10.1136/esmoopen-2018-EACR25.474)

[8] Körner M, Stockli M, Waser B, et al. GLP-1 receptor expression in human tumors and human normal tissues: potential for in vivo targeting. J Nucl Med 2007 48 736–743. (10.2967/jnumed.106.038679) 17475961

[9] Waser B & Reubi JC. Radiolabelled GLP-1 receptor antagonist binds to GLP-1 receptor-expressing human tissues. Eur J Nucl Med Mol Imaging 2014 41 1166–1171. (10.1007/s00259-013-2684-4) 24519555

[10] Perry T, Lahiri DK, Chen D, et al. A novel neurotrophic property of glucagon-like peptide 1: a promoter of nerve growth factor-mediated differentiation in PC12 cells. J Pharmacol Exp Ther 2002 300 958–966. (10.1124/jpet.300.3.958) 11861804

[11] Thompson LD. Pheochromocytoma of the adrenal gland scaled score (PASS) to separate benign from malignant neoplasms: a clinicopathologic and immunophenotypic study of 100 cases. Am J Surg Pathol 2002 26 551–566. (10.1097/00000478-200205000-00002) 11979086

[12] Fishbein L, Leshchiner I, Walter V, et al. Comprehensive molecular characterization of pheochromocytoma and paraganglioma. Cancer Cell 2017 31 181–193. (10.1016/j.ccell.2017.01.001) 28162975 PMC5643159

[13] Tang Z, Kang B, Li C, et al. GEPIA2: an enhanced web server for large-scale expression profiling and interactive analysis. Nucleic Acids Res 2019 47 W556–W560. (10.1093/nar/gkz430) 31114875 PMC6602440

[14] Clark PJ & Evans FC. Distance to nearest neighbor as a measure of spatial relationships in populations. Ecology 1954 35 445–453. (10.2307/1931034)

[15] Ogilvie RF. A quantitative estimation of the pancreatic islet tissue. QJM 1937 6 287–300. (10.1093/oxfordjournals.qjmed.a068286) 13270659

[16] Saisho Y, Butler AE, Manesso E, et al. β-cell mass and turnover in humans: effects of obesity and aging. Diabetes Care 2013 36 111–117. (10.2337/dc12-0421) 22875233 PMC3526241

[17] Kaltsas G, Androulakis II, de Herder WW, et al. Paraneoplastic syndromes secondary to neuroendocrine tumours. Endocr Relat Cancer 2010 17 R173–R193. (10.1677/erc-10-0024) 20530594

[18] Minn AH, Kayton M, Lorang D, et al. Insulinomas and expression of an insulin splice variant. Lancet 2004 363 363–367. (10.1016/s0140-6736(04)15438-x) 15070567

[19] Battocchio M, Zatelli MC, Chiarelli S, et al. Ovarian tumors secreting insulin. Endocrine 2015 49 611–619. (10.1007/s12020-015-0605-y) 25896552

[20] Cascón A, Calsina B, Monteagudo M, et al. Genetic bases of pheochromocytoma and paraganglioma. J Mol Endocrinol 2023 70 e220167. (10.1530/jme-22-0167) 36520714

