# Supplementary material for: Distribution of glucagon-like peptide 1 receptor and insulin in phaeochromocytomas
Source: Endocr Connect. 2026 Jan 13;15(1):e250338. doi: 10.1530/EC-25-0338 (PMC12811711; doi:10.1530/EC-25-0338)
Supplement: Supplementary file 1 [file supplementary_materials.pdf]

Supplementary Table 1 Summary of patients and findings

Patient	Gender/age	Hyperglycaemia*	Tumour size (cm)	PASS$	MN# (nmol/l)	NMN## (nmol/l)	Insulin**	GLP-1R**
1	F/64	+	5.2	0	1.14	13.45	-	-
2	F/32	-	7.5	3	high	high	+	-
3	M/58	+	9.5	4	0.55	2.51	+	+
4	F/50	+	6.0	2	4.17	11.45	+	-
5	M/56	-	3.5	0	2.77	1.01	+	+
6	F/67	-	4.3	0	n	n	+	-
7	F/42	+	2.5	0	0.23	0.92	-	-
8	M/69	-	5.5	2	2.90	2.01	+	+
9	M/45	-	5.0	1	<0.18	1.9	+	-
10	F/55	-	3.0	3	1.72	0.8	+	+
11	F/70	+	2.8	3	1.58	1.67	-	-
12	F/50	-	5.0	4	2.69	2.34	+	+
13	F/-53	+	4.0	0	2.51	10.5	+	-
14	M/5-9	-	5.0	0	0.56	20.22	+	-
15	F/51	-	1.9	0	0.22	2.58	+	-
16	M/43	-	2.3	3	0.19	2.06	+	-
17	M/64	-	2.0	0	0.72	2.32	+	-
18	M/41	-	9.0	4	1.14	11.89	-	+
19	M/36	+	9.5	4	8.27	7.46	-	-
20	M/30	-	6.8	0	3.45	8.41	+	-

* Hyperglycaemia + defined as known diabetes 2 or HbA1c ≥ 48 mmol/mol or fasting blood glucose ≥ 7.0 mmol/l or nonfasting value ≥ 11.1.

$ Pheochromocytoma of the Adrenal Gland Scale Score (PASS) above 4 indicates suspected malignancy.

#Metanephrin (MN), normal plasma upper reference 0.7 nmol/l. High indicates values above upper normal limit. ”n” indicates within normal range.

Normetanephrin (NMN), normal plasma upper reference 1.1 nmol/l. High indicates values above upper normal limit. ”n” indicates within normal range.

**Insulin or GLP-1R (+/-) indicates staining in tumour.
